# Salivary microbiome and hypertension in the Qatari population

**DOI:** 10.1186/s12967-023-04247-8

**Published:** 2023-07-08

**Authors:** Selvasankar Murugesan, Souhaila Al Khodor

**Affiliations:** Maternal and Child Health Division, Research Department, Sidra Medicine, 26999, Doha, Qatar

**Keywords:** 16S ribosomal RNA, Qatar biobank, Saliva, Hypertension, Cardiovascular disease, Qatari population

## Abstract

**Background:**

The prevalence of hypertension in Qatar is 33 percent of the adult population. It is postulated that the salivary microbiome can regulate blood pressure (BP). However, limited investigations exist to prove this hypothesis. Therefore, we examined the difference in the salivary microbiome composition between hypertensive and normotensive Qatari subjects.

**Methods:**

A total of 1190 Qatar Genome Project (QGP) participants (Mean age = 43 years) were included in this study. BP for all participants was classified into Normal (n = 357), Stage1 (n = 336), and Stage2: (n = 161) according to the American Heart Association guidelines. 16S-rRNA libraries were sequenced and analyzed using QIIME-pipeline, and PICRUST was used to predict functional metabolic routes. Machine Learning (ML) strategies were applied to identify salivary microbiome-based predictors of hypertension.

**Results:**

Differential abundant analysis (DAA) revealed that *Bacteroides* and *Atopobium* were the significant members of the hypertensive groups. Alpha and beta diversity indices indicated dysbiosis between the normotensive and hypertensive groups. ML-based prediction models revealed that these markers could predict hypertension with an AUC (Area under the curve) of 0.89. Functional predictive analysis disclosed that Cysteine and Methionine metabolism and the sulphur metabolic pathways involving the renin-angiotensin system were significantly higher in the normotensive group. Therefore, members of *Bacteroides* and *Atopobium* can serve as predictors of hypertension. Likewise, *Prevotella*, *Neisseria,* and *Haemophilus* can be the protectors that regulate BP via nitric acid synthesis and regulation of the renin-angiotensin system.

**Conclusion:**

It is one of the first studies to assess salivary microbiome and hypertension as disease models in a large cohort of the Qatari population. Further research is needed to confirm these findings and validate the mechanisms involved.

**Supplementary Information:**

The online version contains supplementary material available at 10.1186/s12967-023-04247-8.

## Introduction

Hypertension is one of the risk factors for cardiovascular disease (CVD), its prevalence has doubled globally in the last three decades [1]. According to the World Health Organization (WHO), hypertension accounts for 12.8% of all deaths [2]. Factors contributing to hypertension include sedentary lifestyles, unhealthy diets that are high in fat and low in fiber, ethnicity, inappropriate medication use, and stress [3, 4]. Moreover, hypertension can cause damage to the body before symptoms appear, and if left untreated, it can cause several health complications, including coronary heart disease, heart failure, stroke, dementia, kidney failure, etc. [5–7]. Despite a significant progress in the development of antihypertensive medications, efficient dose regimens, and improvements in lifestyle, managing hypertension is still challenging. One out of every five hypertensive patients responds to treatment while the remaining four develop resistance to the treatment [8].

The STEPs-World-Health-Survey revealed that the Qatari population is afflicted by various comorbidities, including obesity (28.8%), high cholesterol (24.7%), and hypertension (14.4%) [9]. In addition, non-communicable diseases are the leading cause of death in Qatar, with a hypertension mortality rate of 6.2 per 100,000 males [10, 11]. Hypertension is diagnosed in approximately 30% of patients aged 25 to 65 years at primary healthcare facilities, and the Stepwise survey indicates that women in Qatar have higher rates of hypertension compared to men [9]. Hence, it is crucial to identify new targets for hypertension diagnosis and personalized treatment.

Saliva is a rich source of proteins, hormones, enzymes, desquamated epithelial cells, and millions of microbes [12, 13]. Although it contains a wealth of resources that can be used to discover biomarkers, most of them remain untapped. Saliva-based biomarkers are highly accessible and non-invasive, making them useful for people of all ages, including infants and the elderly. On the other hand, blood-based biomarkers are invasive and must be sampled by medical personnel.

The cost of sequencing has significantly decreased, and the quality of sequences has improved due to recent advancements [14, 15]. Progress in multi-omics technologies have enhanced our chances to discover novel biomarkers [16–18]. The involvement of the salivary microbiome in maintaining blood pressure homeostasis can be used to explore novel biomarker discoveries in this field. With more than 700 distinct microorganisms, the salivary microbiome is the second most diverse component of the human microbiome following the gut. [19]. Previous studies showed that the core salivary microbiome of healthy subjects includes *Streptococcus, Veillonella, Neisseria,* and *Actinomyces* [20, 21]. Our previously published studies showed that *Bacteroidetes, Firmicutes, Actinobacteria,* and *Proteobacteria* were the common phyla, *Streptococcus, Neisseria, Rothia, Prevotella, Granulicatella, Haemophilus,* and *Porphyromonas* were the dominant genera in the Qatari population [22, 23]. It is worth noting that lifestyle and diet can influence the salivary microbiome composition, which can reflect the host’s health status. This effect can manifest in oral diseases like periodontitis and dental caries, as well as systemic diseases such as diabetes, obesity, cancer, and autoimmune disorders [24–28].

Several studies have been conducted to explore the role of the gut microbiome in hypertension [29–31]. However, despite the salivary microbiome’s accessibility, there is limited research on its involvement in hypertension. The study by Bondonno et al*.* highlighted the importance of the salivary microbiome in hypertension by revealing a disruption in the nitrite-nitrate cycle following the use of antibacterial mouthwash [31]. The study shows that both men and women who used antibacterial mouthwash experienced an increase in blood pressure due to the disruption in the nitric oxide (NO) pathway [31]. In addition, a case–control study examining the relationship between salivary microbiome, hypertension, and salivary NO revealed that subjects with normal blood pressure (BP) had higher NO and more *Neisseria subflava* than those with hypertension [32]. In a recent study, Chen et al. assessed the role of the salivary microbiome in the pathogenesis of obstructive sleep apnea-associated hypertension (OSA-hypertension) and showed that *Haemophilus, Neisseria, Oribacterium,* and *Lautropia* were more enriched in hypertension patients compared to controls [33]. Sohail et al. explored the salivary microbiome diversity changes on a limited sample size (n = 96) of hypertensive Qatari subjects and showed that *Prevotella* and *Veillonella* were significantly higher in the hypertension groups compared to the control group [34].

In this study, we analyzed the salivary microbiome composition of 1190 Qatari participants, randomly selected from the Qatar Genome Project (QGP) cohort. Through the use of machine learning (ML) models, we were able to identify a signature in the salivary microbiome that is associated with elevated blood pressure. This research marks a significant advancement in the development of novel biomarkers that could be used for the diagnosis and treatment of hypertension.

## Results

### Clinical parameters of the study population

From the QBB cohort [23], we randomly selected a total of 1190 Qatari participants. The cohort was classified into four groups based on their blood pressure: Normal BP (n = 336), and three groups for high BP as follows: Elevated (n = 357), Stage1 (n = 336), and Stage2 (n = 161) (Table 1), as described in the “Materials and Methods” section. The mean age of the study participants was 43 years (Table 1), while the normotensive group had a significantly lower average age (34.39 ± 10.12 years) compared to the Elevated (41.63 ± 12.60 years), Stage 1 (46.31 ± 10.27 years), and Stage 2 (52.43 ± 10.14 years) groups (Table 1). Moreover, the BMI, plasma alkaline phosphatase, calcium, cholesterol, glucose, HbA1C, insulin, and urea were significantly higher in the hypertensive groups compared to the normal BP group (Table 1). Particularly Tukey tests for both Cholesterol and insulin levels were performed to observe the significant group with high BP in comparison to Normal group (Additional file 2: Fig S2). The Tukey test of Cholesterol levels inferred that Stage1 group level is significantly higher than normal group (Additional file 2: Fig S2A). On the other hand, the Tukey test of insulin levels confirmed that Elevated, Stage 1 and Stage 2 groups are significantly higher than normal group (Additional file 2: Fig S2B).

**Table 1:** Clinical parameters of the study cohort

	Normal (n = 336)	Elevated (n = 357)	Stage1 (n = 336)	Stage2 (n = 161)	P- value
Age	34.39 ± 10.12	41.63 ± 12.60	46.31 ± 10.27	52.43 ± 10.14	< 0.001^***, a^
Male	220	207	220	78	
BMI (kg/m^2^)	27.38 ± 5.51	31.79 ± 6.05	31.78 ± 4.92	32.52 ± 5.99	< 0.001^***, a^
Systolic BP (mm of Hg)	106.20 ± 7.93	123.56 ± 2.81	130.72 ± 5.79	149.77 ± 10.64	< 0.001^***, a^
Diastolic BP (mm of Hg)	63.86 ± 8.24	70.44 ± 6.89	79.27 ± 8.79	79.96 ± 11.45	< 0.001^***, a^
Albumin(gm/L)	42.57 ± 3.26	44.46 ± 3.45	44.13 ± 3.27	43.13 ± 3.55	< 0.001^***, a^
Alkaline Phosphatase (U/L)	70.91 ± 19.69	76.53 ± 23.22	76.46 ± 26.96	78.01 ± 21.58	< 0.001^***, a^
ALT (GPT) (U/L)	24.72 ± 20.22	27.87 ± 19.49	29.89 ± 20.61	24.6 ± 12.93	< 0.001^***, a^
AST(GOT)(U/L)	22.46 ± 31.66	20.8 ± 8.53	21.79 ± 12.8	19.64 ± 6.81	< 0.001^***, a^
Bicarbonate(mmol/L)	24.66 ± 2.62	25.64 ± 2.56	25.79 ± 2.43	25.83 ± 2.35	< 0.001^***, a^
Calcium (mmol/L)	2.31 ± 0.08	2.3 ± 0.09	2.3 ± 0.1	2.32 ± 0.09	< 0.001^***, a^
Chloride(mmol/L)	102.8 ± 2.13	101.32 ± 2.5	101.26 ± 2.35	100.94 ± 2.66	< 0.001^***, a^
Cholesterol (mmol/L)	4.99 ± 0.95	5.13 ± 1.04	5.25 ± 0.93	5.2 ± 1.18	0.002^**, a^
C-Peptide (ng/mL)	1.91 ± 1.02	2.58 ± 1.56	2.78 ± 1.64	2.88 ± 2.26	< 0.001^***, a^
Creatinine (umol/L)	69.33 ± 11.62	69.24 ± 14.92	70.75 ± 14.47	70.81 ± 20.97	0.315^a^
Vitamin D (ng/mL)	17.42 ± 11.99	18.27 ± 10.69	18.34 ± 10.95	19.98 ± 10.72	0.002^a^
Fibrinogen (gm/L)	3.22 ± 0.66	3.43 ± 0.68	3.42 ± 0.66	3.61 ± 0.74	< 0.001^***, a^
Glucose (mmol/L)	4.73 ± 0.9	5.93 ± 2.29	6.36 ± 2.56	7.59 ± 3.85	< 0.001^***, a^
HBA1C %	5.37 ± 0.8	5.85 ± 1.36	6.06 ± 1.26	6.76 ± 1.85	< 0.001^***a^
HDL (mmol/L)	1.35 ± 0.32	1.28 ± 0.33	1.27 ± 0.36	1.31 ± 0.35	0.002^**a^
Insulin (mcunit/mL)	10.19 ± 9.64	15.96 ± 15.68	16.92 ± 16.1	20.26 ± 27.27	< 0.001^***a^
Iron (umol/L)	16.15 ± 6.72	15.4 ± 5.96	15.93 ± 6.19	14.13 ± 5.65	0.004^*a^
LDL (mmol/L)	3.12 ± 0.9	3.19 ± 0.98	3.22 ± 0.92	3.21 ± 1.14	0.365^a^
Phosphorus (mmol/L)	53.08 ± 9.92	53.02 ± 9.98	52.75 ± 9.52	54.73 ± 8.82	0.25^a^
Potassium (mmol/L)	235.9 ± 71.32	248.48 ± 67.88	238.78 ± 66.64	247.6 ± 67.02	0.111^a^
Sodium (mmol/L)	5.1 ± 0.54	5.15 ± 0.55	5.22 ± 0.59	5 ± 0.57	0.017^*a^
TSH (mIU/L)	12.37 ± 10.86	10.58 ± 9.79	11.08 ± 8.76	8.41 ± 9.16	< 0.001^***a^
Total Protein (gm/L)	68.38 ± 12.32	62.87 ± 10.04	62.13 ± 9.98	63.16 ± 10.09	< 0.001^***a^
Triglyceride (mmol/L)	74.66 ± 4.25	73.87 ± 3.86	73.58 ± 3.68	73.66 ± 3.98	0.003^**a^
Urea (mmol/L)	1.13 ± 0.84	1.48 ± 0.84	1.66 ± 0.97	1.55 ± 0.75	< 0.001^***a^

The parameters mentioned in bold are those significantly elevated in Hypertensive groups (Elevated, Stage 1 and Stage 2) compared to the normotensive group

*BMI* body mass index

^a^Kruskal–Wallis test

^*^Denotes P value < 0.05

^**^P value < 0.01

^***^P- value < 0.001

### Altered salivary microbiome composition and hypertension

**Fig. 1:**
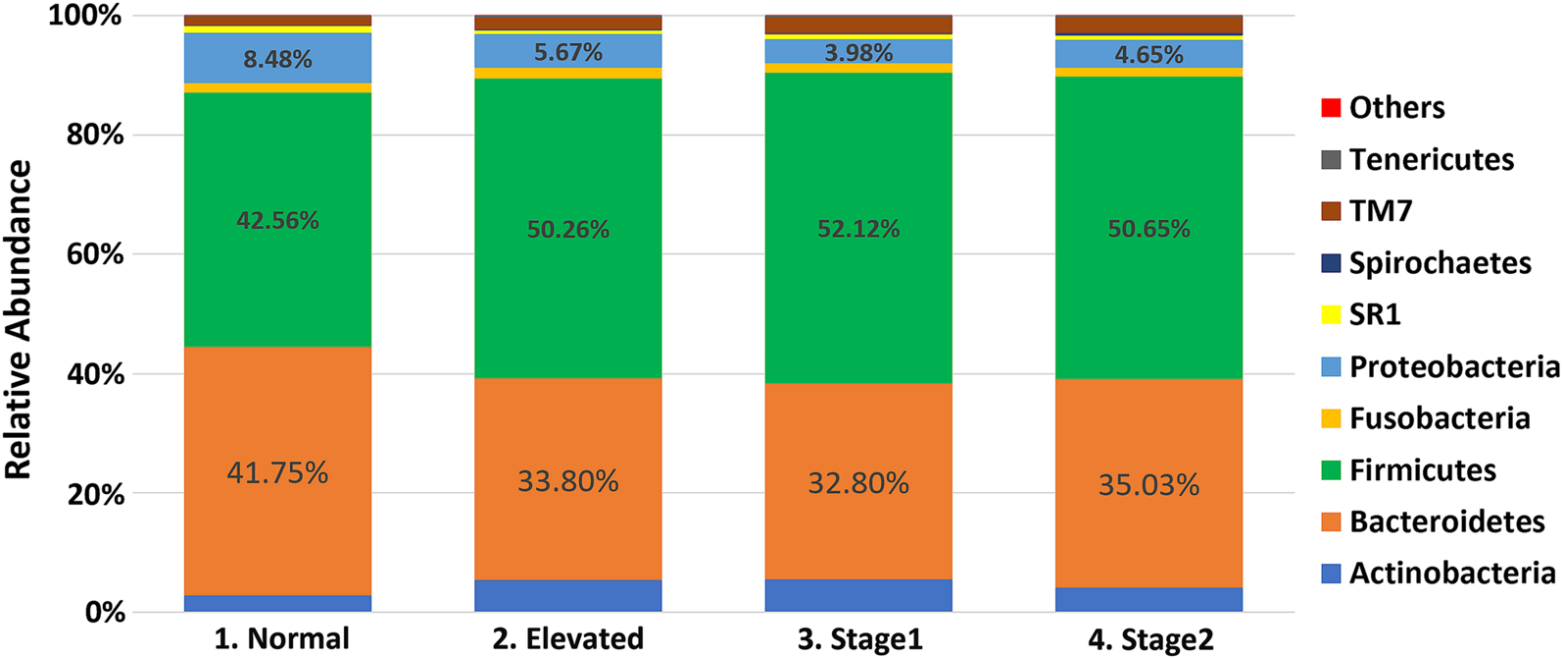
The salivary microbiome composition at the phylum level. Y-axis shows % of relative abundance of the microbiome; X-axis indicates the Normal, Elevated, Stage1, and Stage 2 groups

**Fig. 2:**
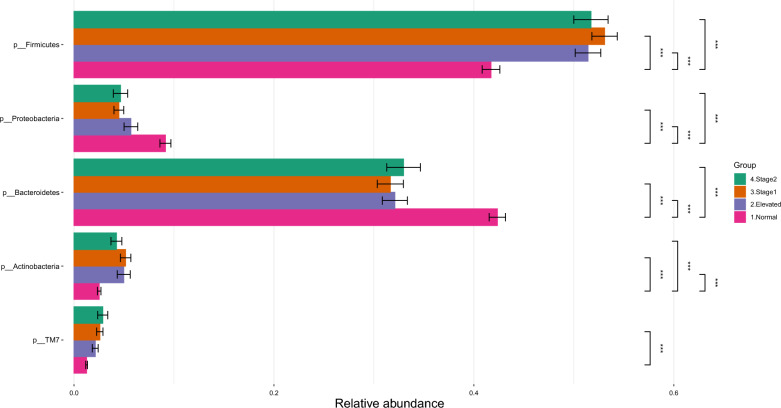
Bar Graphs of differentially abundant salivary microbiome among the phyla. Each color indicates different groups Pink—Normal, Violet—Elevated, Orange—Stage1, Green—Stage 2

**Fig. 3:**
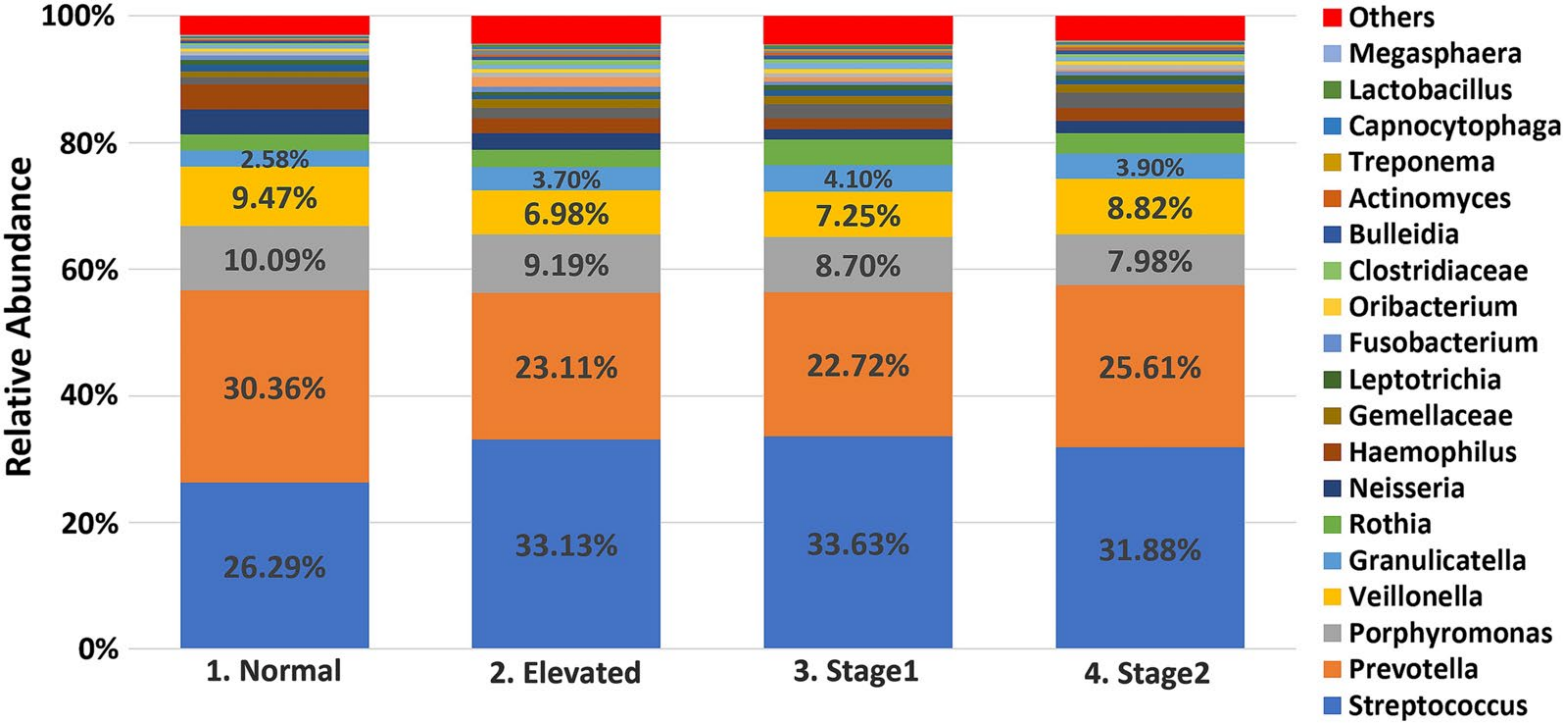
The salivary microbiome composition at the genus level. Y-axis shows % of relative abundance of the microbiome; X-axis indicates the Normal, Elevated, Stage1, and Stage 2 groups

The sequencing of the 16S rRNA amplicons resulted in approximately 48 million reads (47,967,299) for 1190 samples. The median read count per sample was 42,373, the mean was 40,308, and the range varied between 100 and 173,507 sequences. After filtering and alignment, an average of 40,308 assembled reads per sample were assigned to 4813 OTUs. OTUs were classified using the Greengenes bacteria taxonomy and divided into four major phyla: *Bacteroidetes*, *Firmicutes, Actinobacteria*, and *Proteobacteria* (Fig. 1). While the overall composition of the salivary microbiome in all groups was similar, our differential abundance analysis (DAA) of the salivary microbiome at the phylum level revealed that *Bacteroidetes* and *Proteobacteria* were more abundant (*p* < 0.001) in the normal BP group compared to the high BP groups (Fig. 2). Whereas *Firmicutes* and *Proteobacteria* were enriched in the high BP groups (*p* < 0.001) (Fig. 2). At the genus level, our data analysis revealed that the salivary core members were *Streptococcus*, *Prevotella*, *Porphyromonas*, *Granulicatella*, and *Veillonella* (Fig. 3).

**Fig. 4:**
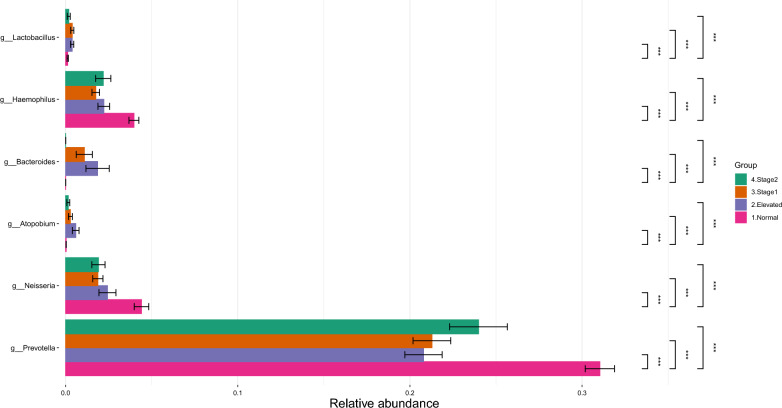
Bar Graphs of differentially abundant salivary microbiome among the genera. Each color indicates different groups Pink—Normal, Violet—Elevated, Orange—Stage1, Green—Stage 2

DAA at the genus level showed that *Prevotella*, *Neisseria,* and *Haemophilus* are significantly higher in the normal BP group compared to the high BP groups (Fig. 4), whereas *Bacteroides, Lactobacillus,* and *Atopobium* are mainly observed in the high BP groups (Fig. 4).

**Fig. 5:**
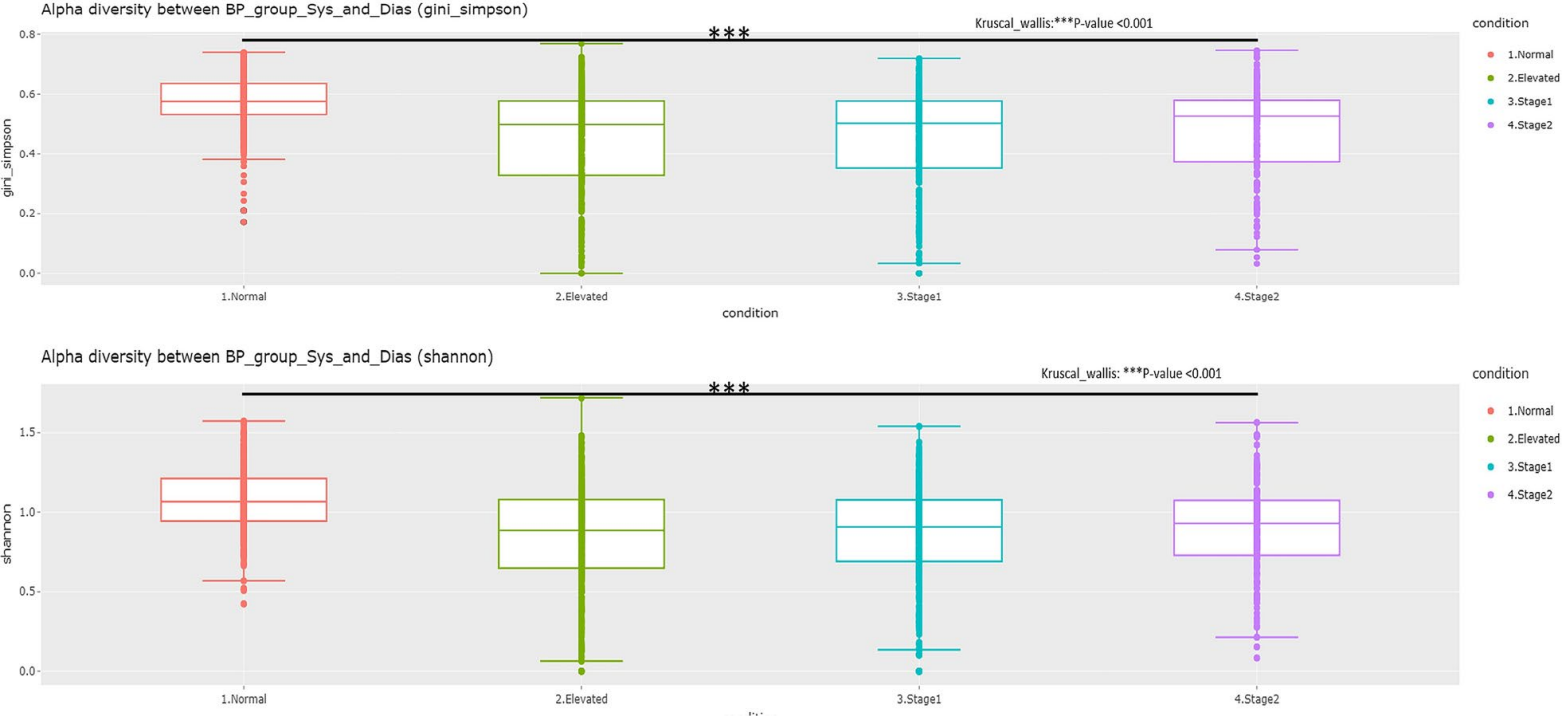
Alpha diversity measures Shannon (top panel) and Simpson (bottom panel) indices for the Normal, Elevated, Stage1, and Stage 2 groups

**Fig. 6:**
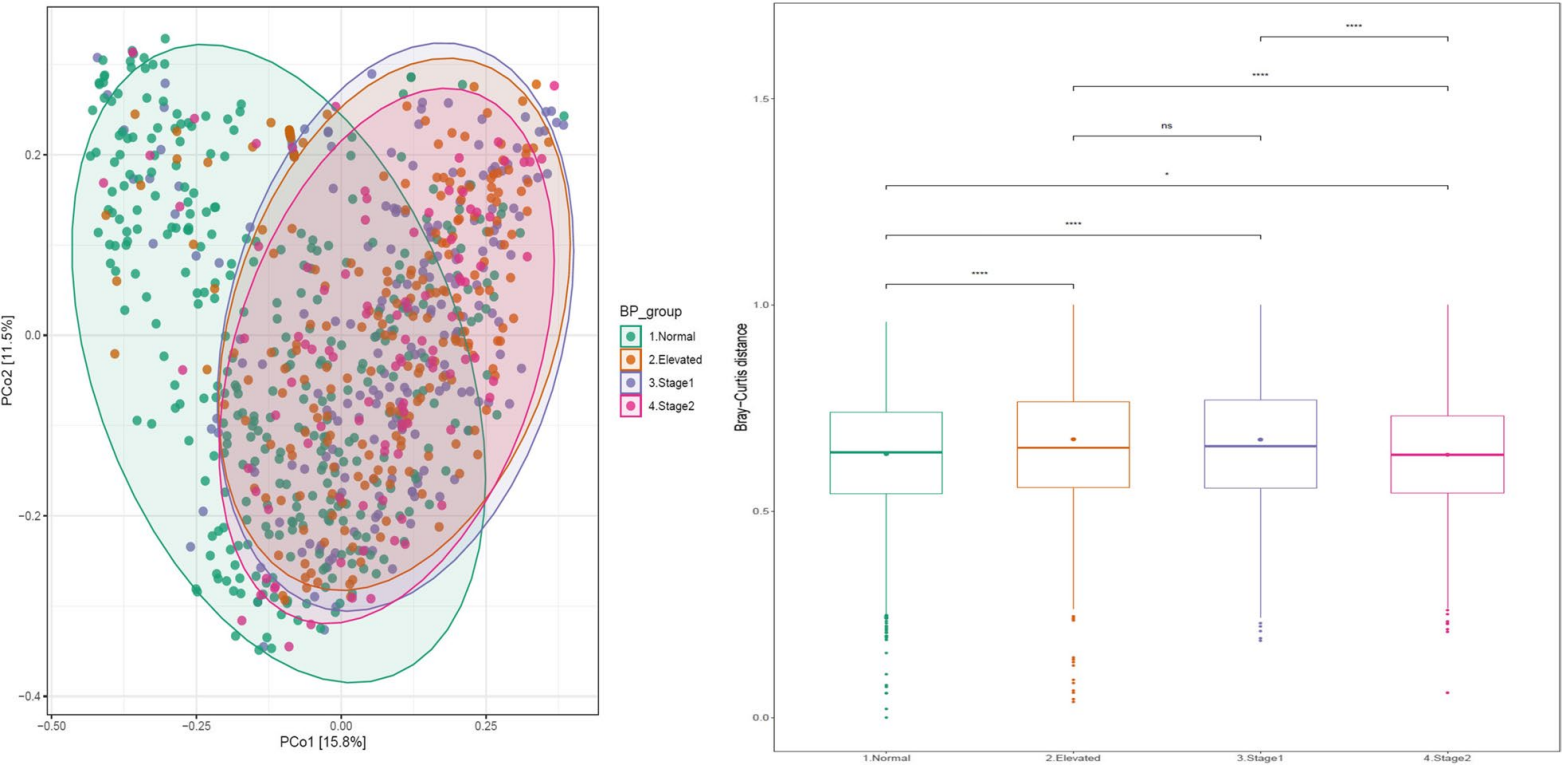
Principal Coordinates Analysis (PCoA) based on Bray–Curtis distances of salivary microbiome. Axes were scaled to the amount of variation explained; Boxplots of bray-distance matrices among the hypertension groups. *P < 0.05, **P < 0.01, ***P < 0.001

The results from the analysis of alpha diversity parameters, specifically the Simpson and Shannon indices, indicate that the normal BP group exhibits significantly higher diversity compared to the high BP groups, as illustrated in Fig. 5. Furthermore, the beta diversity analysis using the Bray–Curtis distance matrix reveals that the normal BP group is significantly different from the high BP groups, as shown in Fig. 6.

### Machine learning approach for predicting hypertension using salivary microbiome

**Fig. 7:**
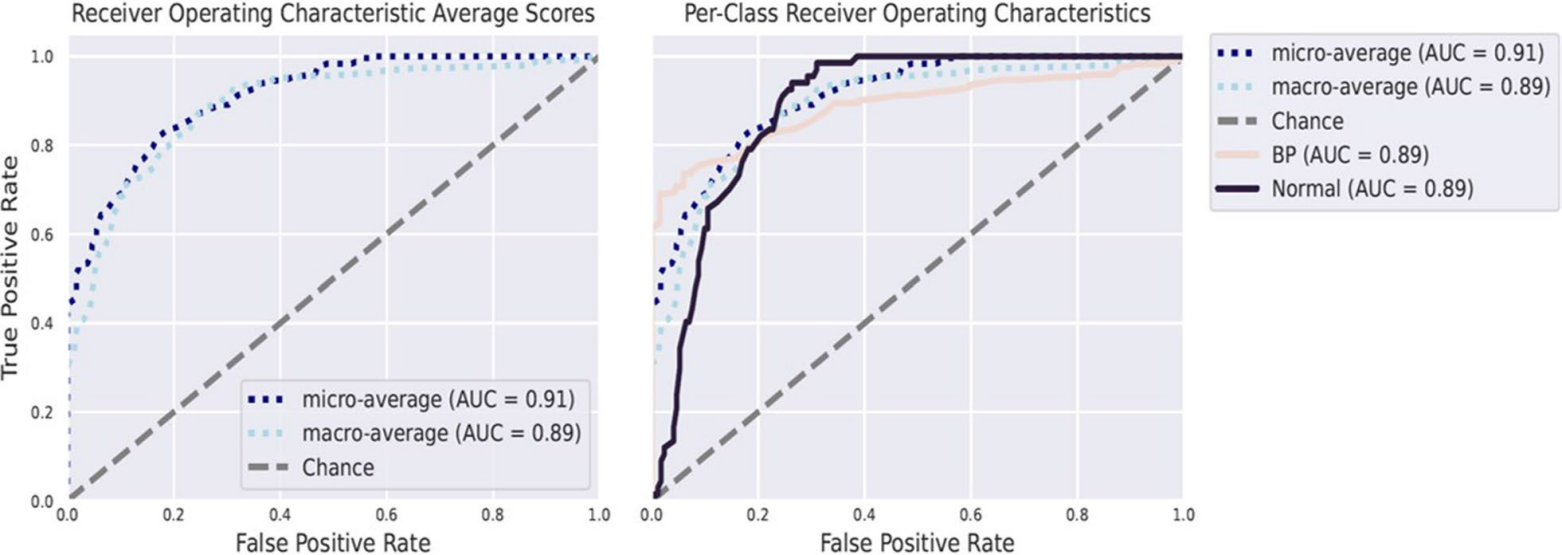
ROC curve for the model of control and BP group, which displayed the cross-validation error as a receiver operating characteristic (ROC) curve with a 95% confidence interval. The area under the ROC (AUROC = 0.89) is given below the curve. The x-axis and y-axis represent false-positive and true-positive rates, respectively, for the tested markers. Pale orange color—BP group; Black color—Normal group. The micro average precision is the sum of all true positives divided by the sum of all true positives and false positives. The macro averaging is the arithmetic means of all recall scores for different groups involved in this study. Macro averaging gives equal weight to each category while micro averaging gives equal weight to each sample

We then applied an independent machine learning (ML) algorithm to distinguish between the salivary microbial communities across the high BP groups and compare them to the normal BP samples. To search for the biomarker validation, we focused on the abundance of six selected bacterial genera including *Prevotella, Neisseria, Haemophilus, Bacteroides, Lactobacillus*, and *Atopobium* as a training set and test set among the 50-random splits of the data. We used Random Forest as the feature estimator as described in the methods section. Our results showed that the selected bacterial markers show a promising area under the curve (AUC) of 0.89 (Fig. 7). Individual group comparisons showed that Normal group has the highest AUC with 0.88, followed by elevated group with 0.67, Stage 2(0.67) and Stage 1 (0.64). Individual AUROC is good (~ > 0.65) with acceptable in comparison with Normal group, but when they are pooled as a single group these identified microbial markers have shown excellent discrimination (Additional file 3: Fig S3).

### Salivary microbial metabolic link with hypertension

**Fig. 8:**
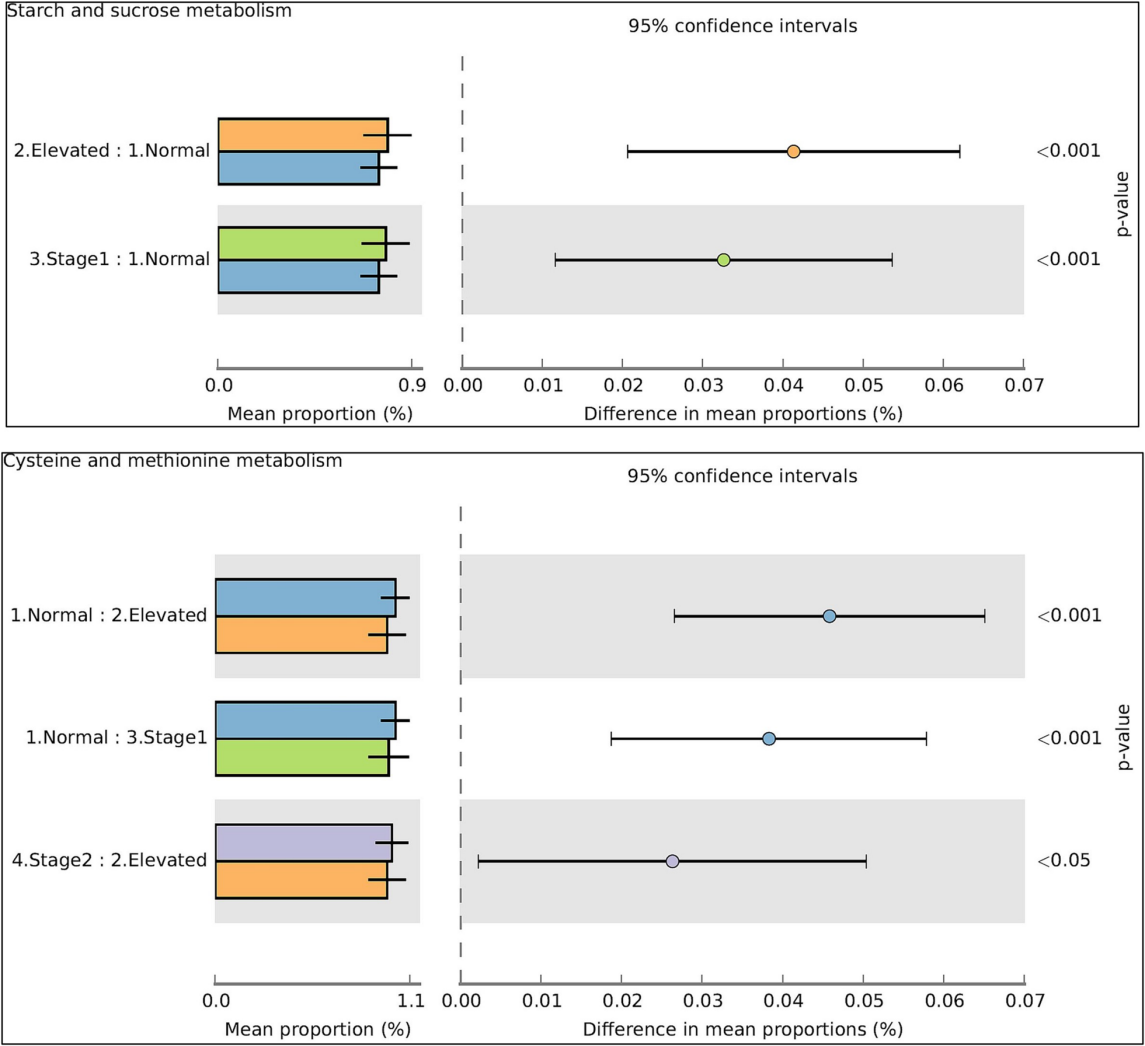
Significant metabolic functional prediction in normal BP group compared to elevated, stage1, and Stage 2 groups (Kruskal Wallis^.^ *P < 0.05;). Blue for normal BP, orange- Elevated, Green for Stage1, violet for Stage 2

**Fig. 9:**
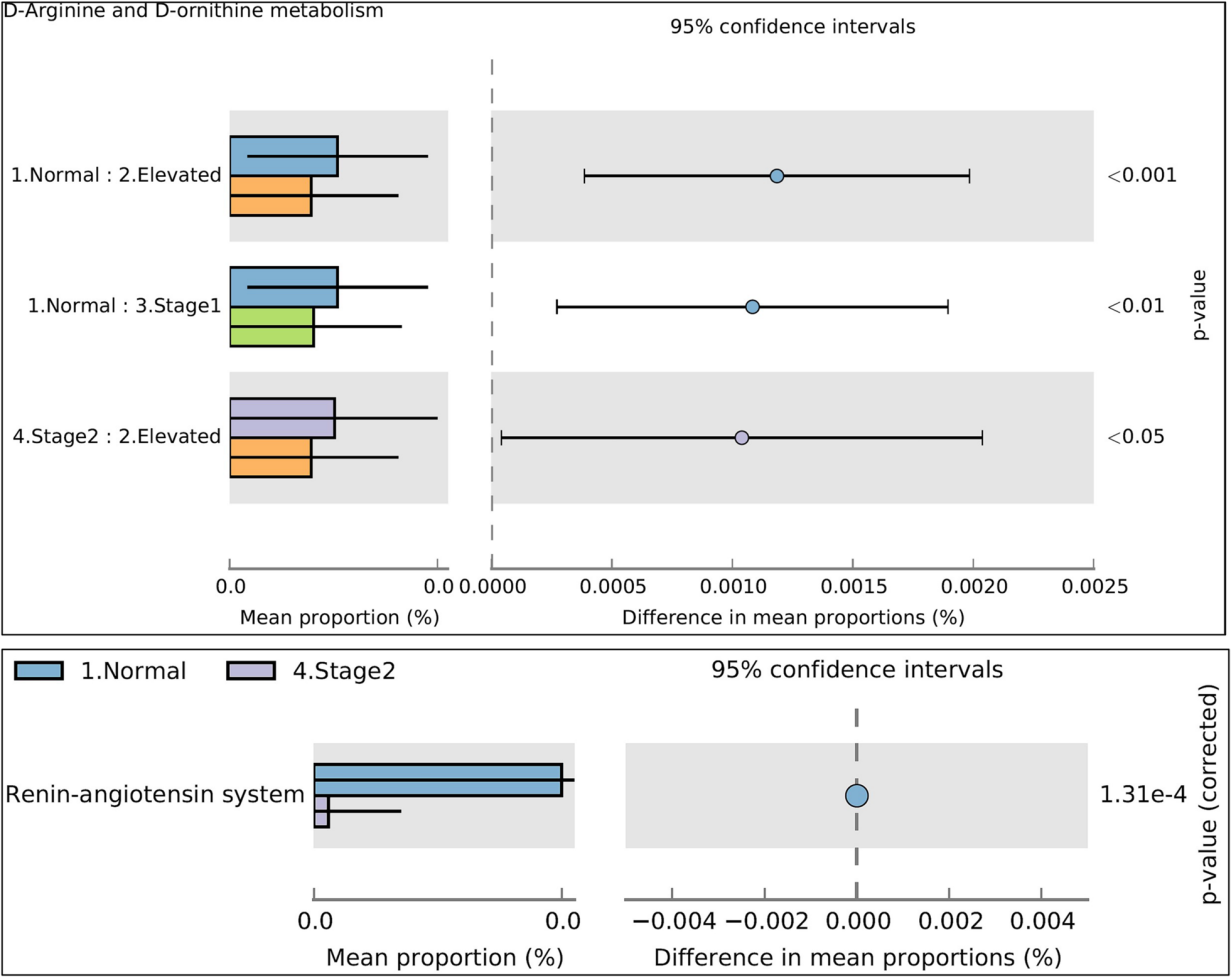
Significant d-Arginine and d-ornithine metabolic and Renin-Angiotensin metabolic functional prediction in normal BP group compared to elevated, stage1, and Stage 2 groups (Kruskal Wallis^.^ *P < 0.05;). Blue for normal BP, orange- Elevated, Green for Stage1, violet for Stage 2

After observing differences in the salivary microbiome profiles across the Normal, Elevated, Stage 1, and Stage 2 groups, we conducted an analysis of the potential functional role of these bacteria in hypertension pathogenesis using PICRUST and KEGG/COG databases. Our analysis revealed significant differences between the estimated functional capabilities of the salivary microbiome in the normal BP group and Elevated, Stage 1, and Stage 2 (Fig. 8). In addition, PICRUSt-KEGG analysis showed a significant increase in the microbes contributing to Starch and sucrose metabolism in the Elevated, Stage 1, and Stage 2 groups than in the normal BP group (Fig. 8). Conversely, microbial sequences linked to sulfur metabolism and sulfur-containing amino acids, including Cysteine and methionine metabolism (Fig. 8), were significantly more abundant in the normal BP group than in the high BP groups. Similarly, predicted metabolic routes of d-Arginine and d-Ornithine metabolism were significantly higher in normal BP compared to elevated and stage 1 groups. In contrast, Conversely, the stage 2 group displayed a notably higher proportion of d-arginine and d-ornithine metabolic pathways compared to the elevated group, as shown in Fig. 9. Additionally, the metabolic pathways for renin-angiotensin system (RAS) were significantly elevated in the normal BP group relative to the stage 2 group (Fig. 9). Moreover, the predicted metabolic pathways using the COG database revealed that COG-4362 (NO synthase) was significantly more abundant in the high BP groups compared to the normal BP group, as depicted in Additional file 1: Fig. S1.

Furthermore, Spearman cross-correlation analysis of COG-4362, selected six salivary microbes, BP values, Cholesterol levels and Insulin levels showed that In normal group, we observed that HDL-cholesterol is positively correlated with C0G-4362 but no significant correlations were observed with neither cholesterol nor insulin levels of high BP groups including elevated, Stage1 and Stage2 (Additional file 4: Fig. S4A–D). Among the bacteria, *Atopobium* showed significantly negative correlation with COG-4362 in elevated, stage1 and stage2 groups, whereas *Neisseria* revealed the significantly positive correlation with COG-4362 in high BP groups.

## Discussion

Hypertension is the third most crucial risk factor for stroke, CVD, and other diseases globally [35]. However, despite the number of hypertension cases increasing worldwide, the mechanism of pathogenesis and effective treatments are still unclear. Previous studies have described the gut microbiome’s role in hypertension in animal and human models [36–38]. However, studies focusing on the salivary microbiome changes during hypertension and their role in its pathogenesis remain sparse. To address this knowledge gap, we conducted a study that analyzed the salivary microbiome of 1190 Qatari subjects participating in QGP. By examining the salivary microbiome, we hope to gain new insights into the relationship between hypertension and changes in the oral microbiome.

In accordance with the American Heart Association (AHA) guidelines, we stratified our cohort based on BP readings into four groups: normal, elevated, stage 1, and stage 2. We found that hypertensive individuals in the latter three groups were older and had a higher body mass index (BMI) compared to normotensive individuals. Though, it is widely reported that HTN is positively linked to increasing age. In this study, the selected participants were age matched with each group (Age range: Normal 19–64; Elevated 18–80; Stage1 19–69; Stage2 19–76) from the cohort of QGP. The dysbiosis is mainly due to an increase in blood pressure irrespective of age factor in this study. In Chinese population, obstructive sleep apnea patients with comorbid HTN showed dysbiosis of salivary microbiome than healthy control with same age group [33]. A metanalysis of 4 cohorts study confirms that increased tendency HTN observed in women than men in their third decade of the life [39]. Another observational study infers that specific oral microbes are associated with the baseline BP and increased risk of HTN in menopausal women [40]. Based on these literature evidences, we infer that HTN and SM are associated/linked irrelevant of age factor. Our vascular system in addition, hypertensive individuals also had higher levels of C-peptide, HbA1C, glucose, and insulin and higher cholesterol levels than normotensive individuals. These findings are not surprising, as many studies have established a strong association between metabolic syndrome, which is characterized by a cluster of abnormal metabolic conditions such as obesity, diabetes, and hyperlipidemia [41, 42] and hypertension.

In our study cohort, we found that alkaline phosphatase (ALP) levels were significantly higher in the high BP groups than the normal BP group. ALP is a clinical marker of bone or hepatic diseases and is typically derived equally from the liver and bone in healthy individuals [43]. Previous investigations have suggested that increased levels of ALP may be associated with vascular calcification, which may play a significant role in the development of vascular disease. Furthermore, previous studies indicated that cerebral small artery dysfunction and CVD are both associated with greater serum ALP levels [44–46].

Numerous studies have explored the potential role of the gut microbiome in the pathophysiology of various diseases such as diabetes, obesity, and hypertension, among others [47–50]. However, studies on the salivary microbiome have been conducted at a much lower level, and there have been very few studies on the role of the salivary microbiome in hypertension [23, 34, 51, 52].

In our previous studies, we examined the salivary microbiome and found that *Bacteroidetes, Firmicutes,* and *Proteobacteria* were the predominant phyla, and *Streptococcus, Prevotella, Porphyromonas,* and *Veillonella* were the most common genera among the Qatari population [22, 23, 53]. These findings underscore the need for further research to better understand the potential role of the salivary microbiome in hypertension and other diseases.

In our current study, we explored the diversity and microbial changes in the saliva of Qatari participants suffering from hypertension. Our results revealed that subjects with high BP (Elevated, Stage 1, and Stage 2) have significantly lower diversity in their salivary microbiome compared to those with normal BP. This reduction in microbial diversity was previously reported in subjects using chlorhexidine mouthwash and has been shown to positively correlate with increased BP by altering the abundance of nitrate-reducing bacteria [54–56]. Furthermore, we found that *Prevotella, Neisseria,* and *Haemophilus* were significantly enriched in the normal BP group compared to the other groups, which are the most abundant microbial members of saliva and essential oral nitrate-reducing bacteria [57, 58] to regulate BP in normal group. A comparative study between hypertensive and normotensive participants showed that *Prevotella* is considerably elevated in the normotensive group [59, 60]. A case–control study that assessed the link between salivary NO, hypertension, and the microbiome showed that *Neisseria subflava* and salivary NO were significantly higher in normotensive when compared to hypertensive subjects [32]. Our findings suggest that *Haemophilus* and *Neisseria* are essential oral nitrate-reducing bacteria that regulate systemic BP via the nitrate-nitrite-NO pathway [61, 62]. A dysbiosis or reduction of these critical salivary bacteria that regulate BP may promote endothelial dysfunction and increase the risk of CVD.

On the other hand, Qatari participants with high BP displayed a notable increase in the abundance of *Atopobium, Bacteroides,* and *Lactobacillus*. Sohail et al. previously showed that *Atopobium* was significantly overrepresented in the hypertensive group [34]. Yan et al. also reported that *Bacteroides* were significantly more abundant in the hypertensive group compared to controls [50]. Similarly, Silveira-Nunes et al. showed that *Lactobacillus* is significantly more prevalent in the Brazilian hypertensive cohort [63].

We employed the random forest classifier, a supervised machine learning algorithm, to investigate whether the microbial signature we found between the groups can serve as biomarkers for hypertension. The classification models using six microbial features that were used together yielded an area under the receiver operating curve (AUC) value of 0.89 in the sensitivity–specificity plot. Our study is the first to predict the BP-associated salivary microbial marker using a Machine learning approach in the Qatari population. A cohort of hypertensive patients will be needed to further validate our findings.

PICRUSt-KEGG analysis revealed that the predictive microbial metabolic functions such as starch, and sucrose metabolism were increased in the hypertensive groups and that cysteine and methionine metabolism, as well as the sulfur metabolisms, were increased in the normal BP group. High starch and sucrose metabolic routes in hypertension groups suggest that those microbes will have a higher ability to extract more carbohydrates from the diet when present in the oral cavity and later convert the excess sugar into lipids [64]. Hypertension, obesity, dyslipidemia and insulin resistance are the factors positively associated with each other [65]. The body's extra calories will cause cellular deaths of visceral adipocytes and be engulfed by macrophages to form crown-like structures [66]. In addition, it induces the expression of TNF-Alfa and IL-6, and nitric oxide synthase [67]. These compound changes might provide its pathophysiological association with hypertension, insulin resistance, and dyslipidemia. It is also well-known that sulfur metabolism is involved in the metabolism of sulfur-containing amino acids such as cysteine and methionine to regulate the arterial blood pressure [68]. PICRUSt-COG analysis revealed that the microbial clusters of orthologs such as COG 4362 (Nitric Oxide Synthase) were significantly higher in BP than in the normal groups. Nitric oxide synthase metabolizes arginine to produce Nitric Oxide, which regulates blood pressure through angiotensin-II [69]. Negative correlation with LDL and COG4362 indicates its regulatory role to reduce the BP and CVD risk in normotensive group. In contrast, showed significantly negative correlation with COG-4362 in elevated, stage1 and stage2 groups. An imbalance in this cycle will lead to oxidative stress-mediated endothelial dysfunction. Our findings may provide insight into the role of salivary bacteria and their role in hypertension pathophysiology and progression.

## Conclusions

Associations of salivary biomarkers with hypertension were assessed using a combination of 16S rRNA gene sequencing, in silico prediction, and ML-based models. Developing an early screening/treatment model for hypertension is essential to provide better healthcare for our patients. The salivary microbiome significantly influences host health through its involvement in many physiological and biological pathways. A profound understanding of this complex dynamic structure might improve our understanding of diseases and advance their diagnosis. In summary, our data show that the salivary microbiome composition was significantly different between the normal, elevated, stage1, and stage 2 hypertension groups, including *Haemophilus, Prevotella*, and *Neisseria*, which were found to be enriched in the normal BP group.

On the other hand, *Bacteroides* and *Lactobacillus* were enriched in the high BP group and were predicted to increase carbohydrate metabolic routes. *Prevotella, Haemophilus,* and *Neisseria* may act as protectors to regulate BP via nitric acid synthesis and regulation of the renin-angiotensin system. More experiments using in vitro and in vivo models are needed to confirm our findings and validate those mechanisms.

## Methods

### Study cohort

The study was approved by the Institutional Review Board (IRB) of Sidra Medicine under (protocol #1510001907) and by Qatar Biobank (QBB) (protocol #E/2018/QBB-RES-ACC-0063/0022. All experiments were performed under the approved guidelines. QBB and Sidra Medicine signed a collaboration agreement to collect coded saliva samples along with phenotypic and clinical data. In this study, we analyzed samples and data from 1190 Qatari participants who were randomly selected from the Qatari Genome Project (QGP). All participants were 18 years old and above, and no exclusion criteria were applied. There were 725 males and 465 females included (Table 1). Anthropometric and blood parameters were analyzed for each participant, including BMI, total protein, hemoglobin, albumin, ferritin, calcium, iron, vitamin D, cholesterol, HDL, LDL, triglycerides, and glucose levels.

Following the American heart association guidelines [70], the study cohort was categorized based on their blood pressure readings. The categories included Normal BP (normotensive) which is defined as having blood pressure of less which was defined as having a systolic blood pressure between 120 and 129 mm Hg and a diastolic blood pressure of less than 80 mm Hg; Stage 1, which was defined as having a systolic blood pressure between 130 and 139 mm Hg or a diastolic blood pressure between 80 and 89 mm Hg; and Stage 2, which was defined as having a systolic blood pressure of at least 140 mm Hg or a diastolic blood pressure of at least 90 mm Hg. The Mann–Whitney test was used to calculate statistical significance using MINITAB-17 [71]. *P*-values less than 0.05 were considered statistically significant.

### Total salivary DNA extraction

Saliva samples were collected in QBB as described previously [22], and stored at − 80 °C until further analysis. Then, the total salivary DNA was extracted using the automated QIAsymphony protocol (Qiagen, Hilden, Germany), following the manufacturer's instructions [23].

### 16S rRNA gene sequencing and data analysis

The V1–V3 regions of the 16S rRNA gene were amplified using the Illumina Nextera XT library preparation kit (FC-131-1002). The amplified PCR products of ~ 650 bp in size from each sample were purified using Agencourt AMpure XP magnetic beads (Beckman Coulter) and pooled in equimolar concentrations. High throughput sequencing was performed on an Illumina MiSeq 2 × 300 PE (Illumina, Inc. San Diego) in accordance with manufacturer's instructions. Image analysis and base calling were carried out directly on the MiSeq. The sequence data were analyzed using QIIME1.9.0 pipeline [22, 72]. Operational taxonomic units (OTUs) were generated by aligning against the Greengenes database (Version:13_8) with a confidence threshold of 97% [73].

### Taxonomic and diversity analyses

The relative abundance of the salivary microbiome of the study groups was generated using R- "MicroEco" package [74] from the OTUs generated using QIIME. Differential abundant analyses of salivary microbiome among the study groups at the phylum and genus levels were done by univariate – Wilcoxon test using the same package. Alpha diversity measures, including Shannon, and Simpson indices, were calculated with "animalcules" package [75]. Beta diversity indices were presented as principal coordinate analysis and the differences in the Bray–Curtis distance matrix between the study groups was performed using MicroEco [74].

### Prediction of metabolic routes and functional differences among the groups

The metagenome KEGG orthologs (KOs), clusters of orthologs groups (COGs) and RNA families (Rfam) [76] of the analyzed samples were predicted with the Phylogenetic Investigation of Communities by Reconstruction of Unobserved States (PICRUSt) tool [77] against the OTUs present in the Greengenes database [73]. The detected KOs were then collapsed to the pathway level (KEGG level 3) using PICRUSt. The profiles of functional pathways were further analyzed with Kruskal Wallis and Tukey–Kramer post hoc analysis. These were then corrected for multiple testing with the Bonferroni method using the software package statistical analysis of taxonomic and functional profiles (STAMP) [78].

### Machine learning modeling

The salivary microbiome biomarkers were predicted using a supervised learning classifier based on hypertension. We randomly split the data 50-times into a training set (80%) on which the predictive models were built and a test set (20%) on which we tested the performance of each model. Optimal tuning parameters were chosen via fivefold cross-validation. The test set validated the classification accuracy of the Random Forest as an optimized estimator [79].

## Supplementary Information

**Additional file 1: Figure S1.** Significant clusters of orthologous genes (COGs) in normal BP group compared to elevated and stage1 (Kruskal Wallis. *P < 0.05;). Blue for normal BP, orange- Elevated, Green for Stage1.

**Additional file 2: Figure S2.** ANOVA with Tukey’s multiple comparisons test was used to determine statistically significant differences between the BP groups in comparison with normotensive group (*P < 0.05, **P< 0.01). Means that do not share a letter are significantly different.

**Additional file 3: Figure S3.** ROC curve for the model of Control, Elevated, Stage1, and Stage2 groups, which displayed the cross-validation error as a receiver operating characteristic (ROC) curve with a 95% confidence interval. The area under the ROC (AUROC = 0.89) is given below the curve. The x-axis and y-axis represent false-positive and true-positive rates, respectively, for the tested markers. Pale orange color—Normal group; Pale maroon—Elevated; Maroon—Stage 1 and Black color indicates Stage2 groups.

**Additional file 4: Figure S4.** Correlation analysis between selected six salivary microbes, COG-4362, Cholesterol, insulin and BP in the study groups. **A** Normal **B** Elevated **C** Stage1 **D** Stage2. Red to blue color scale indicates a positive to a negative correlation, respectively. * P<0.05, **P<0.01, ***P<0.001

## Data Availability

The datasets presented in this study can be found in online repositories. The names of the repository/repositories and accession number(s) can be found below: https://www.ncbi.nlm.nih.gov/PRJNA781451.
