# Supplementary figures and images for: Salivary microbiome and hypertension in the Qatari population
Source: J Transl Med. 2023 Jul 8;21:454. doi: 10.1186/s12967-023-04247-8 (PMC10329805; doi:10.1186/s12967-023-04247-8)

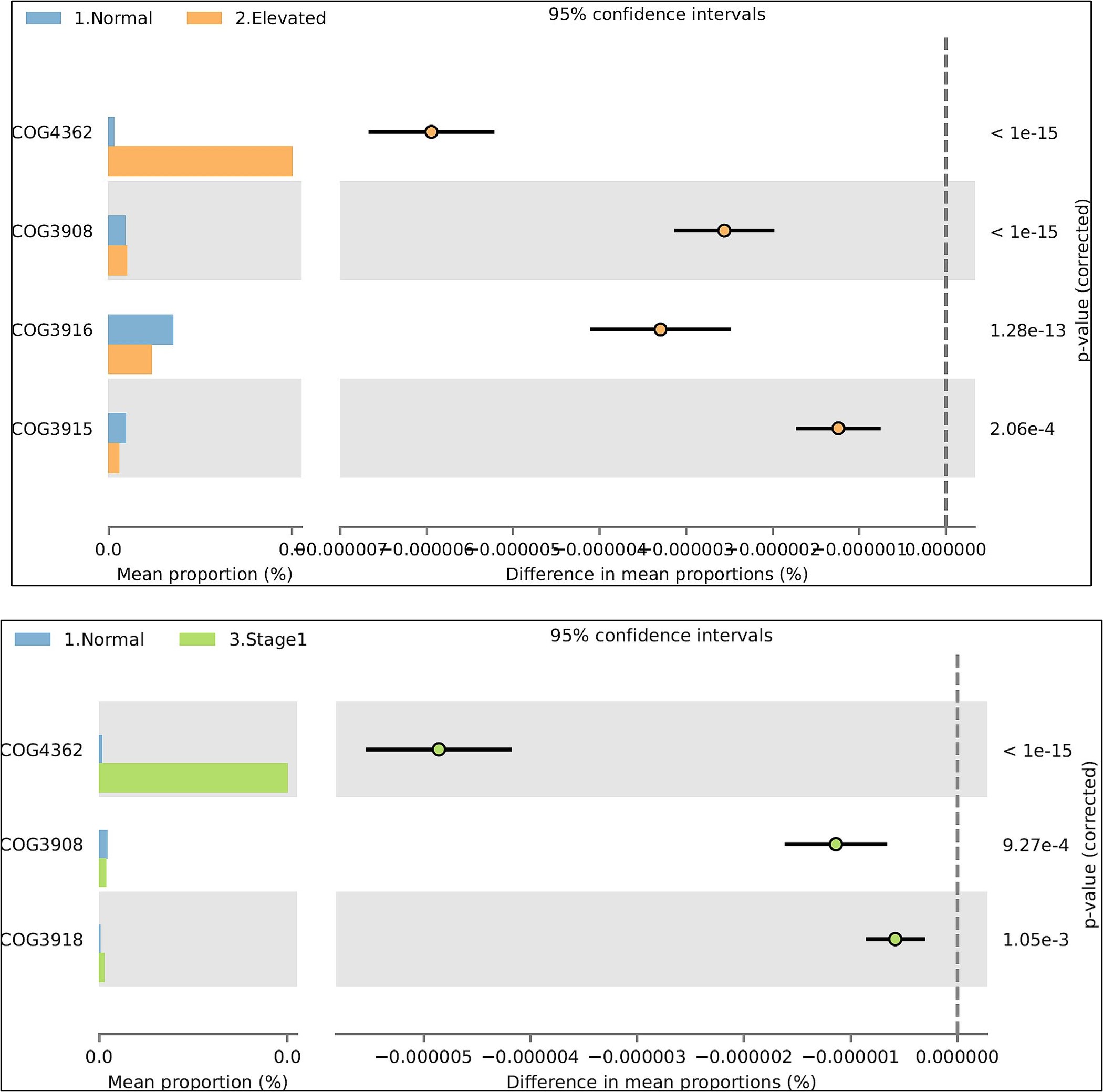

Supplement: Supplementary file 1 — Additional file 1: Figure S1. Significant clusters of orthologous genes (COGs) in normal BP group compared to elevated and stage1 (Kruskal Wallis. *P < 0.05;). Blue for normal BP, orange- Elevated, Green for Stage1. [file 12967_2023_4247_MOESM1_ESM.jpg]

# A) NORMAL

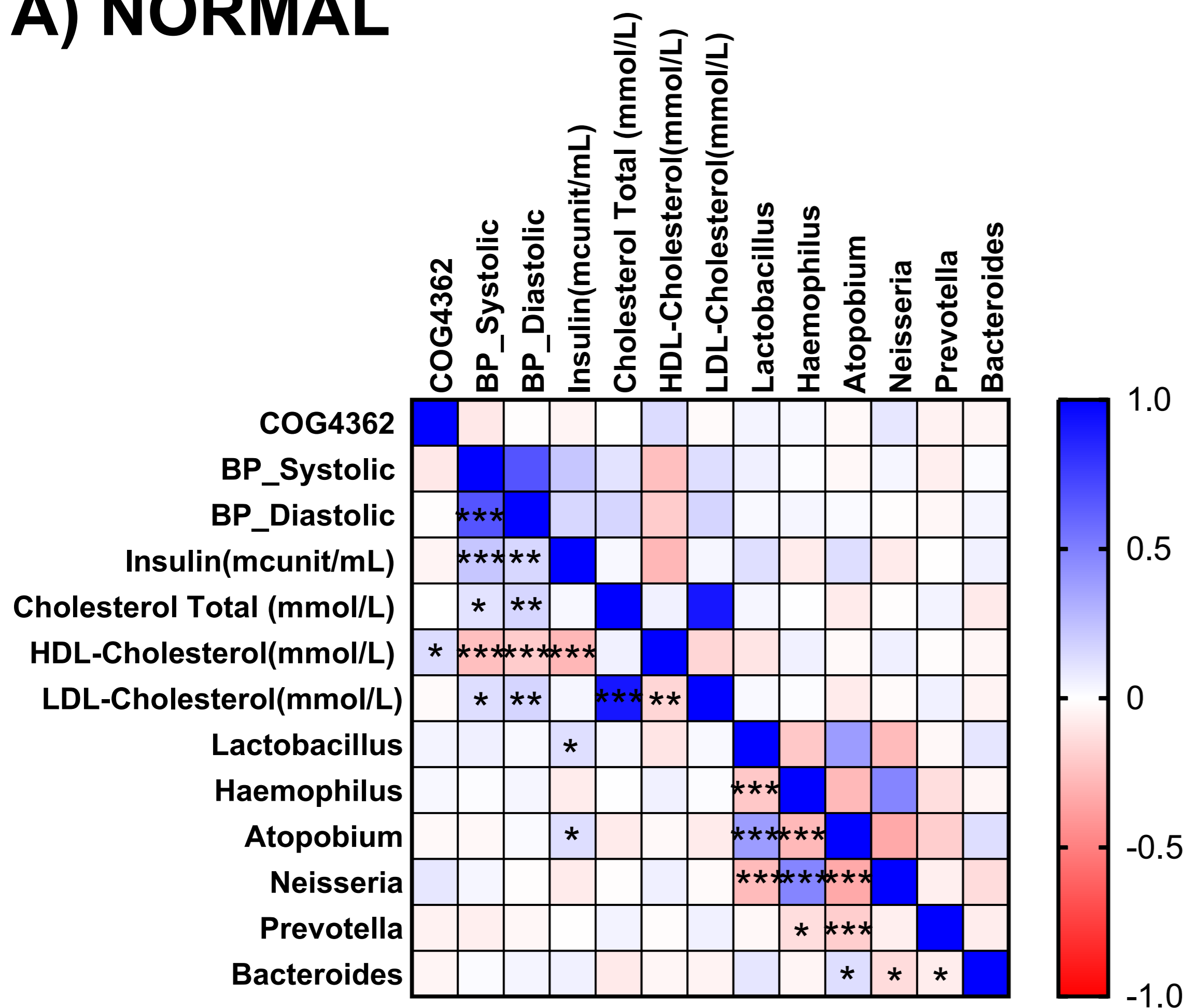

# B) ELEVATED

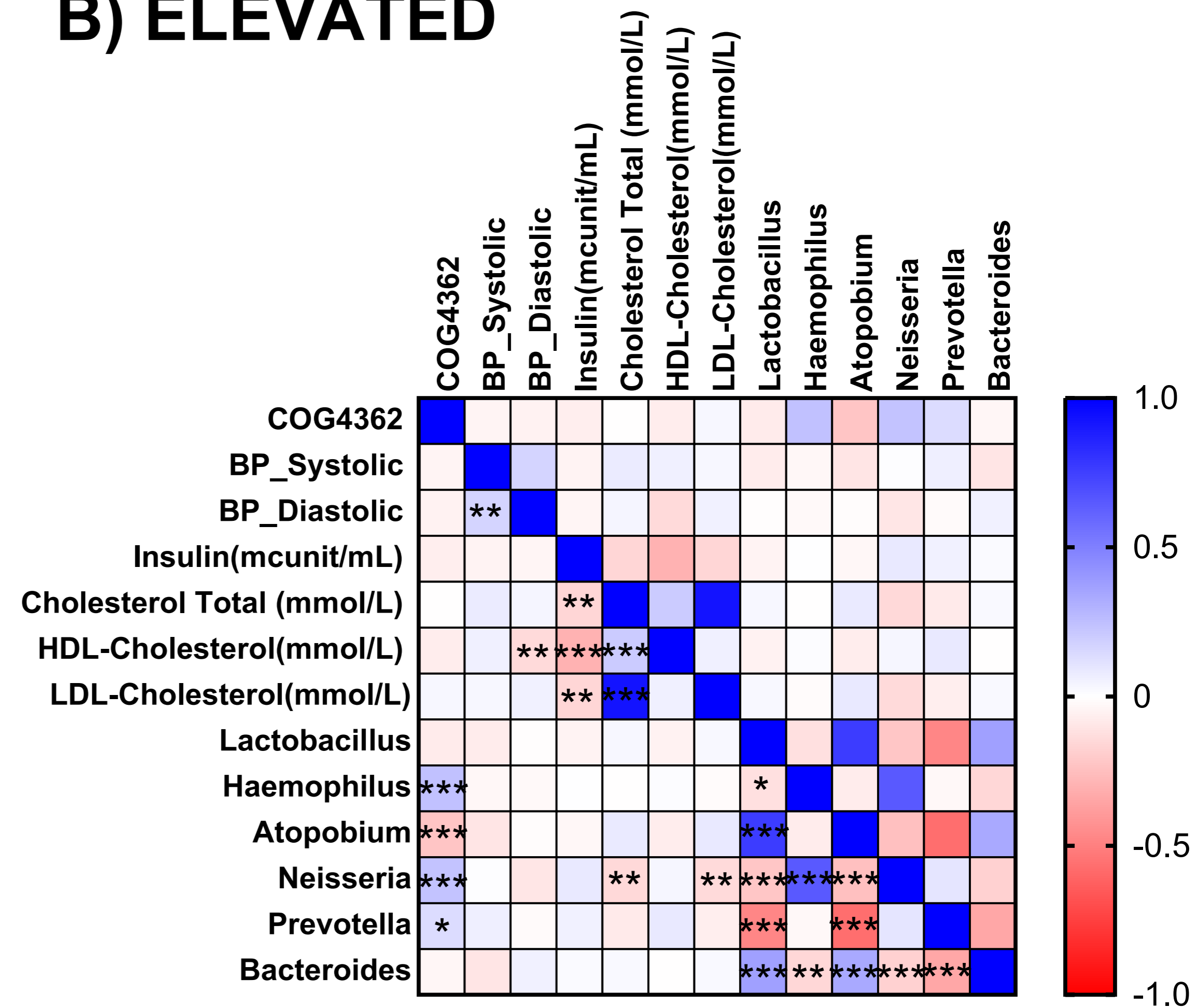

# C) STAGE 1

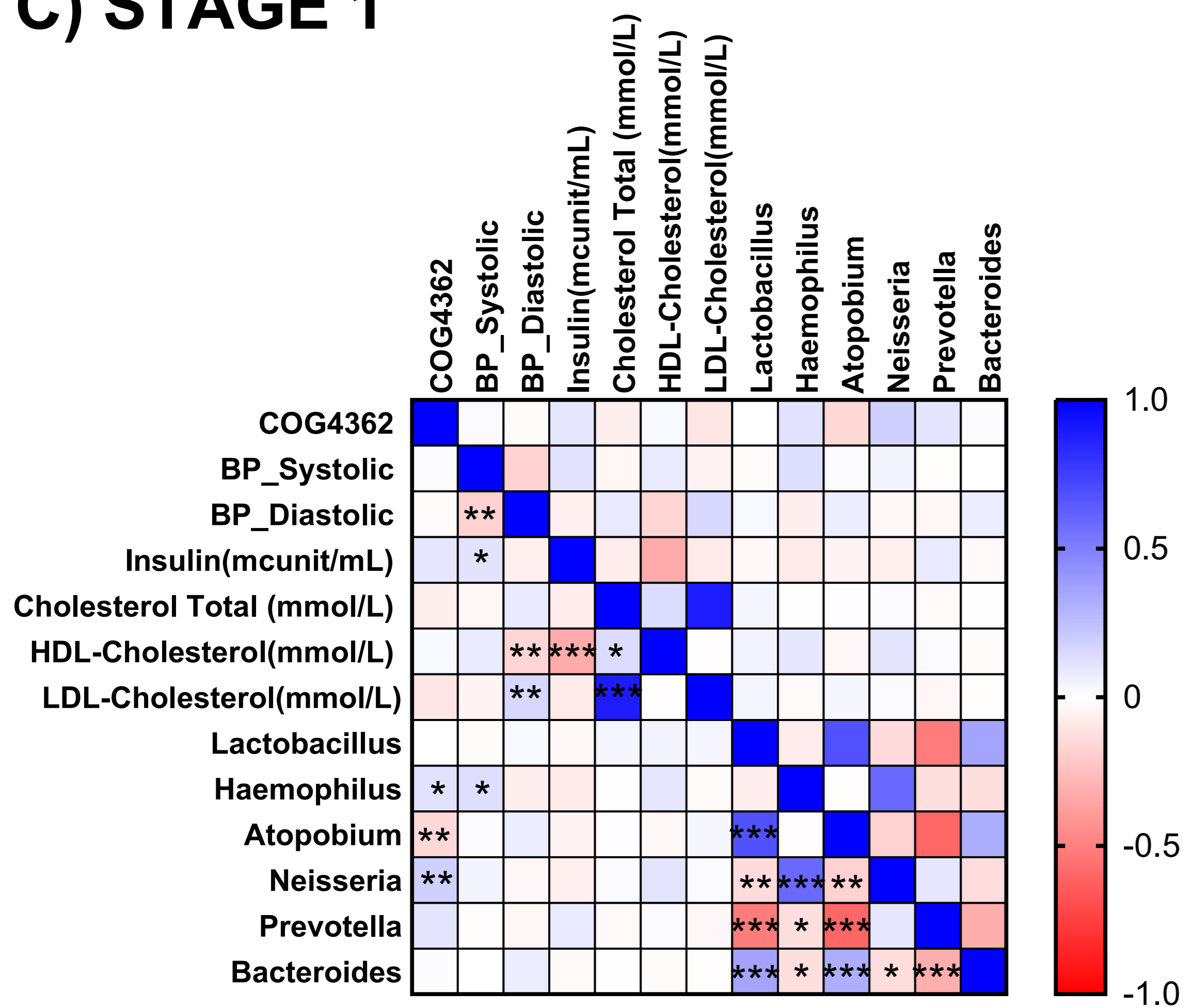

# D) STAGE 2

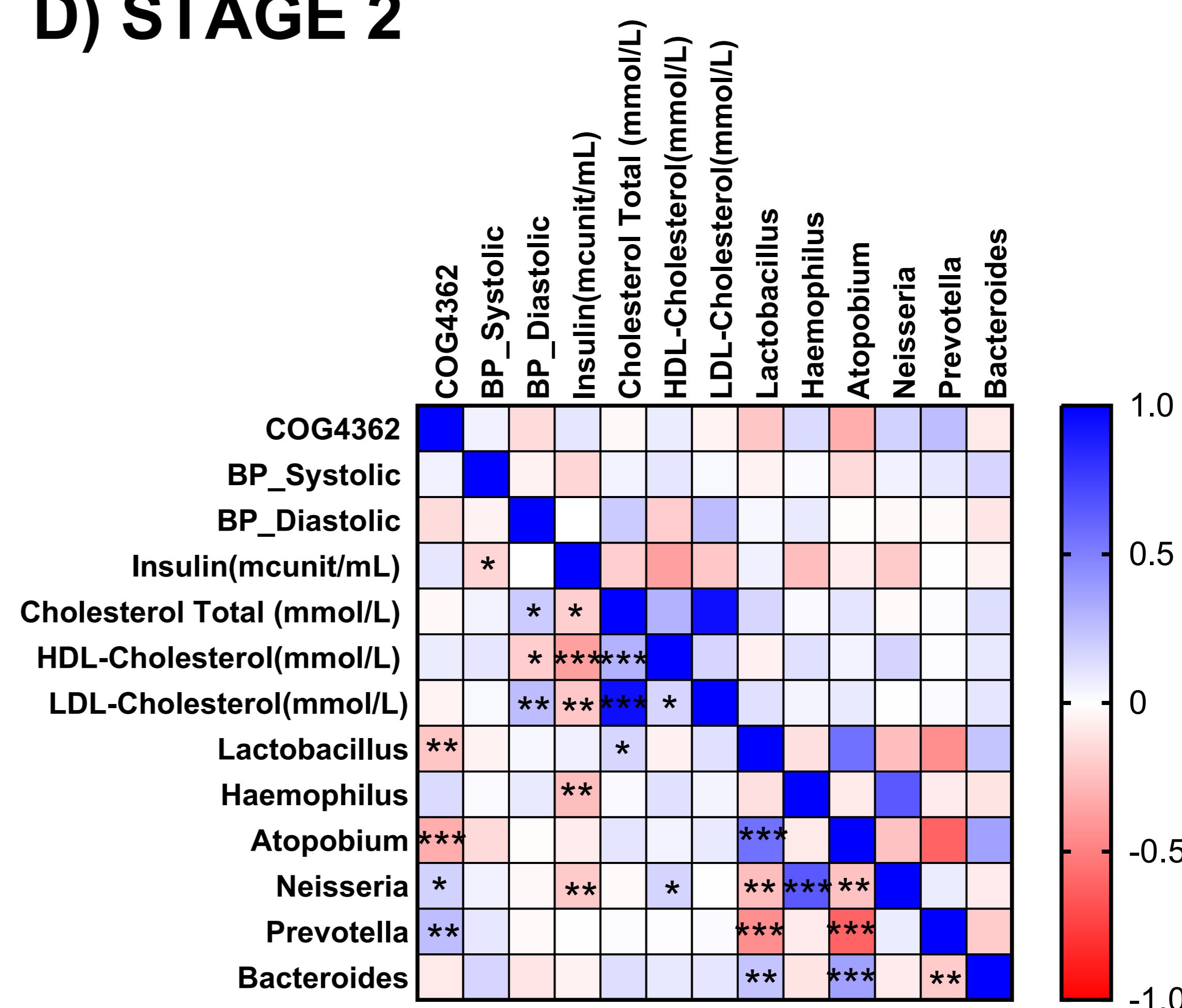

Supplement: Supplementary file 4 — Additional file 4: Figure S4. Correlation analysis between selected six salivary microbes, COG-4362, Cholesterol, insulin and BP in the study groups. A Normal B Elevated C Stage1 D Stage2. Red to blue color scale indicates a positive to a negative correlation, respectively. * P<0.05, **P<0.01, ***P<0.001 [file 12967_2023_4247_MOESM4_ESM.pdf]
