# Supplementary material for: Salivary microbiome and hypertension in the Qatari population
Source: J Transl Med. 2023 Jul 8;21:454. doi: 10.1186/s12967-023-04247-8 (PMC10329805; doi:10.1186/s12967-023-04247-8)
Supplement: Supplementary file 2 — Additional file 2: Figure S2. ANOVA with Tukey’s multiple comparisons test was used to determine statistically significant differences between the BP groups in comparison with normotensive group (*P < 0.05, **P< 0.01). Means that do not share a letter are significantly different. [file 12967_2023_4247_MOESM2_ESM.pdf]

A)

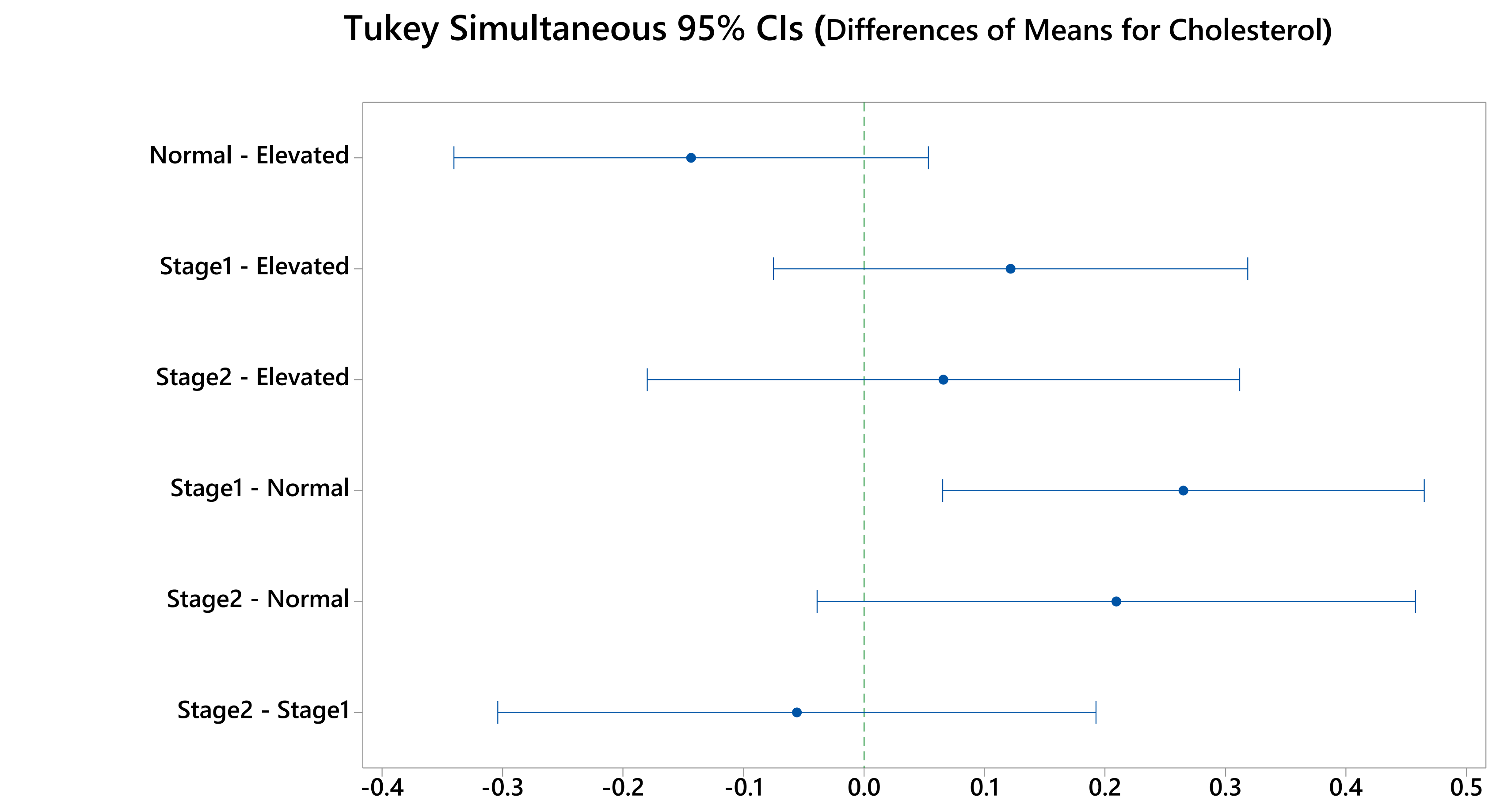

Means

BP_group	N	Mean	StDev	95% CI
Elevated	357	5.1322	1.0438	(5.0274, 5.2370)
Normal	336	4.9887	0.9495	(4.8807, 5.0967)
Stage1	336	5.2539	0.9347	(5.1459, 5.3619)
Stage2	161	5.1981	1.1867	(5.0421, 5.3542)

Pooled StDev = 1.00917

Tukey Pairwise Comparisons

Grouping Information Using the Tukey Method and 95% Confidence

BP_group	N	Mean	Grouping
Stage1	336	5.2539	A
Stage2	161	5.1981	A B
Elevated	357	5.1322	A B
Normal	336	4.9887	B

Means that do not share a letter are significantly different.

B)

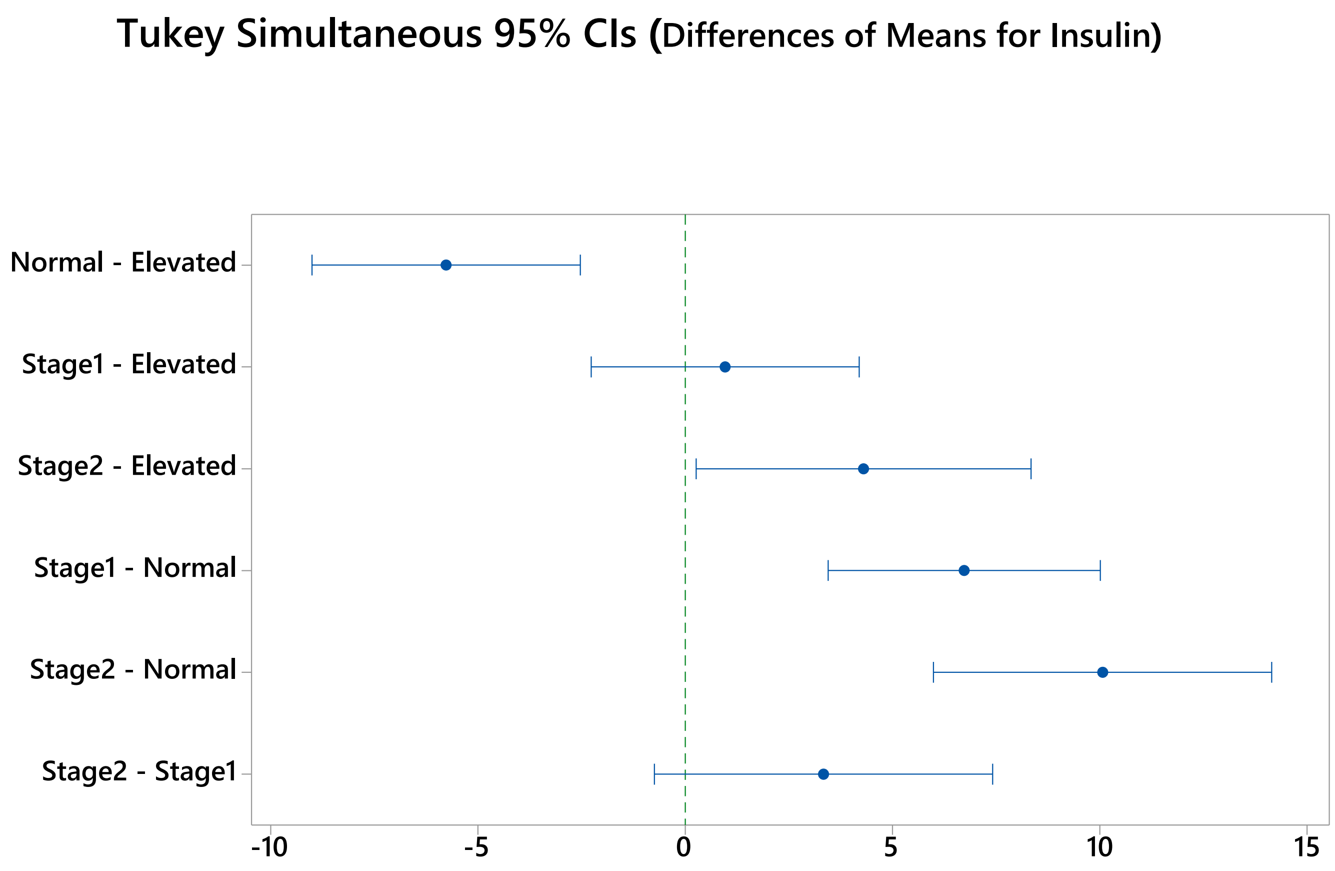

Means

BP_group	N	Mean	StDev	95% CI
Elevated	357	15.958	15.705	(14.237, 17.680)
Normal	336	10.191	9.658	(8.417, 11.966)
Stage1	336	16.922	16.127	(15.148, 18.696)
Stage2	161	20.26	27.35	(17.70, 22.82)

Pooled StDev = 16.5762

Tukey Pairwise Comparisons
Grouping Information Using the Tukey Method and 95% Confidence

BP_group	N	Mean	Grouping
Stage2	161	20.26	A
Stage1	336	16.922	A B
Elevated	357	15.958	B
Normal	336	10.191	C

Means that do not share a letter are significantly different.
