# Supplementary material for: Salivary microbiome and hypertension in the Qatari population
Source: J Transl Med. 2023 Jul 8;21:454. doi: 10.1186/s12967-023-04247-8 (PMC10329805; doi:10.1186/s12967-023-04247-8)
Supplement: Supplementary file 3 — Additional file 3: Figure S3. ROC curve for the model of Control, Elevated, Stage1, and Stage2 groups, which displayed the cross-validation error as a receiver operating characteristic (ROC) curve with a 95% confidence interval. The area under the ROC (AUROC = 0.89) is given below the curve. The x-axis and y-axis represent false-positive and true-positive rates, respectively, for the tested markers. Pale orange color—Normal group; Pale maroon—Elevated; Maroon—Stage 1 and Black color indicates Stage2 groups. [file 12967_2023_4247_MOESM3_ESM.pdf]

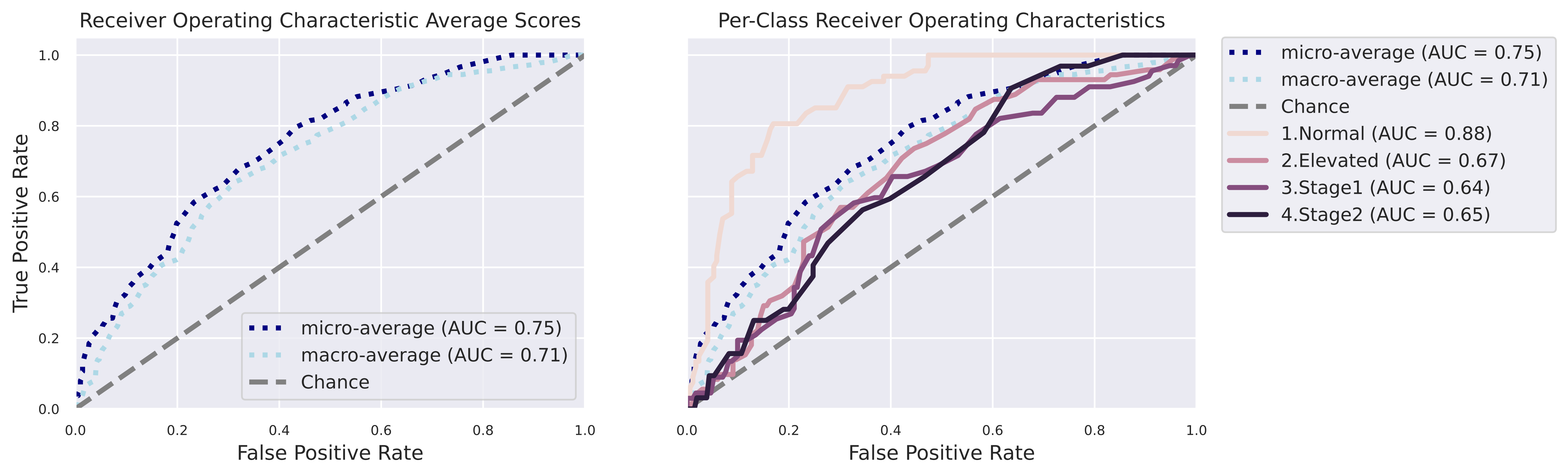

Receiver Operating Characteristic Average Scores
True Positive Rate
False Positive Rate
0.0
0.2
0.4
0.6
0.8
1.0
micro-average (AUC = 0.75)
macro-average (AUC = 0.71)
Chance
Per-Class Receiver Operating Characteristics
False Positive Rate
micro-average (AUC = 0.75)
macro-average (AUC = 0.71)
Chance
1.Normal (AUC = 0.88)
2.Elevated (AUC = 0.67)
3.Stage1 (AUC = 0.64)
4.Stage2 (AUC = 0.65)
